# When does structure modeling go wrong? A PDB-scale analysis of protein structure model validation using DAQ Score

**DOI:** 10.1063/4.0000975

**Published:** 2025-10-27

**Authors:** Daisuke Kihara, Tsukasa Nakamura, Genki Terashi

**Affiliations:** 1 Purdue University, West Lafayette, IN, USA; 2 KEK, Tsukuba, Ibaragi, Japan

## Abstract

Errors in structure modeling occur probably more frequently than one might think in cryo-EM structure determination, particularly when the map resolution is not very high. To this end, we have developed DAQ (Deep-learning-based Amino acid-wise model Quality) score, a machine learning-based method for detecting structural outliers in protein models. DAQ employs deep neural networks to analyze local density features of amino acids and atoms, assessing the likelihood of correct residue modeling (Terashi et al., *Nat. Methods*, 2022).

Here, we present a large-scale analysis of over 10,000 protein structure models from the Protein Data Bank (PDB), revealing common trends in modeling errors. Our findings provide insights into systematic inaccuracies and guide improvements in structure validation. To facilitate broader accessibility, DAQ assessment results are available in the DAQ-DB database (https://daqdb.kiharalab.org) and integrated into PDBj (Nakamura et al., *Nat. Methods*, 2023). Furthermore, we introduce DAQ-Refine, an automated refinement protocol designed to correct errors detected by DAQ (Terashi et al., *Acta Cryst. D*, 2023).

